# Vulnerable victims in court: from childhood to senescence

**DOI:** 10.1080/07853890.2021.1896199

**Published:** 2021-09-28

**Authors:** Iris Almeida, Ana Filipa Fernandes, Catarina Frade, Carolina Nobre, Lúcia Osório

**Affiliations:** a Instituto Universitário Egas Moniz (IUEM), Egas Moniz Cooperativa de Ensino Superior, Caparica, Portugal; b Centro de Investigação Interdisciplinar Egas Moniz (CiiEM), Egas Moniz Cooperativa de Ensino Superior, Caparica, Portugal; c Laboratório de Psicologia Egas Moniz (LabPSI-EM), Centro de Investigação Interdisciplinar Egas Moniz (CiiEM), Egas Moniz Cooperativa de Ensino Superior, Caparica, Portugal; d Laboratório de Ciências Forenses e Psicológicas Egas Moniz (LCFPEM), Centro de Investigação Interdisciplinar Egas Moniz (CiiEM), Egas Moniz Cooperativa de Ensino Superior, Caparica, Portugal; e Gabinete de Informação e Atendimento à Vítima – Espaço Cidadania e Justiça (GIAV – Egas Moniz/DIAP Lisboa), Lisboa, Portugal

## Abstract

**Introduction:**

The trial process is usually stressful for victims. Testifying in criminal court must cope potentially traumatising effects and implies recalling and evoking a crime. Such act tends to be a revictimization [1–3]. Several studies have shown that re-traumatization of victims in criminal justice process has serious problems [3].The article 271 of the Portuguese Code of Criminal Procedure allows the prosecutors and the inquiring judge to record the victim’s testimony and use it during the future trial. This procedure, called future memory statements, prevents the victim to be exposed months or years later to the memories of a traumatic event [2]. Even this procedure has a traumatic outcome due to the normal anxiety felt by victims. To minimise this it is necessary to inform and prepare the victims for this diligence, explaining the court procedures. Prepare the vulnerable victim for future memory statements aims to avoid an emotional trauma event towards the criminal justice system. The purpose of this paper is to demonstrate the work developed by the Victims Information and Assistance Office (GIAV), and its role as technical advisor to the Public Prosecutor's Office, specifically about victim’s attendance.

**Materials and methods:**

GIAV was established in partnership with Egas Moniz Higher Education School and it is located at the Combat Unit against Domestic Violence, 7^th^ Section of the Lisbon Public Prosecutor’s Office. Data was collected between March 2013 and May 2015 from future memory statements protocol that comprises three phases: Pre-inquiry stage (1 or 2 days before inquiry), inquiry stage (trial hearing) and post-inquiry stage (after trial hearing) [1,2]. A total of 144 statements for future memory were performed by GIAV. All ethical issues have been taken due to the sensitive nature of the involved data involved and the respective informed consentient, the confidentiality limits, and information about the ethics and technician’s impartiality.

**Results:**

The type of crime of vulnerable victims is mostly child sexual abuse (*n* = 55, 39.5%), child abuse and neglect (*n* = 42, 29.9%) and domestic violence (*n* = 26, 18.8%). The other type of crime are sexual harassment (*n* = 7, 4.9%), intimate partner violence (*n* = 4, 2.1%), and exposure to violence (*n* = 2, 1.4%), rape (*n* = 2, 1.4%) and others (e.g. threats and sexual coercion, crimes against freedom). We evaluate 98 female victims and 42 male victims, aged between 4 and 79 years old (*M* = 14.06, *SD* = 11.32).The relationship between victims and defendants are: 55 sons and daughters, 49 others situations, mostly more than one defendant (like parent and stepfather, grandmother and uncle), 16 stepchild, 6 ex-girlfriend and others (neighbour, grandchild, unknow).

**Discussion and conclusions:**

It can be seen that the statements for future memory diligence is properly present in the current reality, and it can be concluded that the use of a structured protocol allows to benefit the quantity and quality of information, promoting, within what is a particularly vulnerable victim experience, making it very effective. At the same time, it allows the articulation between law, criminal justice system and forensic psychology, allowing an effective management of the process, praising the superior interest of the victim as well as emotional stability. Victim’s statements are crucial to our criminal justice system and give us a better understand of the experience of testifying and how we can reduce re-traumatization and revictimization of victims.
